# Determinants of job satisfaction among laboratory workers

**DOI:** 10.1192/j.eurpsy.2026.11813

**Published:** 2026-07-31

**Authors:** I. Sellami, A. Abbes, A. Feki, D. Elleuch, M. L. Masmoudi, M. Hajjaji, K. Jmal Hammami

**Affiliations:** 1 occupational medicine; 2 Rheumatoloy, Hedi Chaker Hospital; 3 Family department, Medecine university, sfax, Tunisia

## Abstract

**Introduction:**

Job satisfaction is a key psychological construct reflecting workers’ attitudes toward their work and organizational environment. Identifying its determinants is crucial, as job satisfaction can significantly influence both productivity and overall performance among laboratory workers.

**Objectives:**

This study aimed to study the determinants of job satisfaction among laboratory workers.

**Methods:**

A descriptive, analytical, cross-sectional survey was conducted among laboratory workers. Data were collected on sociodemographic and occupational characteristics. Job satisfaction was rated on a 0–10 scale, where 0 indicated low and 10 indicated high satisfaction. Participants reported their height and weight, which were used to calculate Body Mass Index (BMI). Perceived stress was assessed using the 4-item Perceived Stress Scale (PSS-4).

**Results:**

The study included 91 laboratory workers with a mean age of 44.2 ± 9.2 years. The majority were women (92.2%, n = 83), and 80% were married. On average, participants had 14.8 ± 9.5 years of work experience. The mean job satisfaction score was 6.5 ± 2.6. the mean BMI was 26 ±7.1, and the mean perceived stress score was 6.3 ± 2.6. Job satisfaction was significantly higher among participants with lower BMI and lower perceived stress (p < 0.05).

**Conclusions:**

Job satisfaction among laboratory workers was influenced by both BMI and perceived stress, with lower stress levels and healthier BMI associated with higher satisfaction. These findings underscore the importance of promoting physical health and stress management to enhance workplace well-being and performance.

**Disclosure of Interest:**

None Declared

